# Development of a Highly Sensitive Immuno-PCR Assay for the Measurement of α-Galactosidase A Protein Levels in Serum and Plasma

**DOI:** 10.1371/journal.pone.0078588

**Published:** 2013-11-13

**Authors:** Sachie Nakano, Yoshihito Morizane, Noriko Makisaka, Toshihiro Suzuki, Tadayasu Togawa, Takahiro Tsukimura, Ikuo Kawashima, Hitoshi Sakuraba, Futoshi Shibasaki

**Affiliations:** 1 Department of Molecular Medical Research, Tokyo Metropolitan Institute of Medical Science, Tokyo, Japan; 2 Synthera Technologies Co., Ltd., Tokyo, Japan; 3 Department of Analytical Biochemistry, Meiji Pharmaceutical University, Tokyo, Japan; 4 Department of Functional Bioanalysis, Meiji Pharmaceutical University, Tokyo, Japan; 5 Department of Clinical Genetics, Meiji Pharmaceutical University, Tokyo, Japan; University of Houston, United States of America

## Abstract

Fabry disease is an X-linked genetic disorder caused by defects in the α-galactosidase A (*GLA*) gene, and heterogeneous mutations lead to quantitative and/or qualitative defects in GLA protein in male patients with Fabry disease. Random X-chromosomal inactivation modifies the clinical and biochemical features of female patients with Fabry disease. Functional polymorphisms have been frequently reported in recent times, and these increase the difficulty of understanding the pathogenetic basis of the disease. To date, GLA protein level has been measured using an enzyme-linked immunosorbent assay (ELISA). However, ELISA is not highly sensitive due to the high background noise. In this paper, we introduce a novel application of the immuno-polymerase chain reaction (PCR) method (termed Multiple Simultaneous Tag [MUSTag]) for measurement of the GLA protein level in blood samples. We compared the sensitivities of the MUSTag method with plates or magnetic beads with those of ELISA for recombinant human GLA and found that the apparent maximal sensitivity was higher for the former than for the latter. We then measured the GLA concentrations in serum and plasma from male patients with classic Fabry disease (Male Fabry), females with Fabry disease (Female Fabry), male subjects harboring the functional polymorphism p.E66Q (E66Q), and control (Control) subjects. Our results revealed that compared to the MUSTag plate and ELISA, the MUSTag beads assay afforded a clearer estimation of the GLA protein levels in the serum and plasma with minimal or no background noise, although all the methods could differentiate between the Male Fabry, E66Q, and Control groups. The Female Fabry group showed characteristic heterogeneity, which was consistent with the X-linked inheritance. This novel method is expected to be useful for the sensitive determination of GLA level in blood and elucidation of the pathogenetic basis of Fabry disease.

## Introduction

Human α-galactosidase A (GLA, EC 3. 2. 1. 22) is a lysosomal hydrolase involved in the catabolism of α-d-galactosyl (GLA) conjugates. Deficiency in GLA activity causes systemic accumulation of glycolipids, predominantly globotriaosylceramide (Gb3), and leads to the X-linked genetic disorder Fabry disease (OMIM 301500) [1]. Male patients with classic Fabry disease typically present with pain in the peripheral extremities, angiokeratoma, hypohidrosis, and corneal clouding during childhood or adolescence and develop renal, cardiac, and cerebrovascular involvement with increasing age, although some patients with the late-onset type of the disease develop heart and kidney disorders without the childhood symptoms. Heterozygous female patients with Fabry disease exhibit heterogeneous clinical presentations, ranging from asymptomatic to severe disease due to random X-chromosomal inactivation.

Fabry disease is usually diagnosed by the measurement of the GLA activity, detection of glycolipid accumulation, histopathological examination, and genetic analysis. To date, more than 600 different causative gene mutations have been identified for Fabry disease (Human Gene Mutation Database, http://www.hgmd.cf.ac.uk/). Among them, gross alterations, nonsense mutations, and most of the splicing mutations of the *GLA* gene lead to a deficiency of the GLA protein, but missense mutations comprising the majority of mutations cause heterogeneous pathogenesis, i.e., some of them affect the active site, while others decrease the stability of the enzyme molecule [2], [3]; thus, the molecular basis of Fabry disease is complex. Recently, functional polymorphisms, such as p.E66Q [4], [5] and p.D313Y [6], have been frequently found in Japanese/Korean and Caucasian populations, respectively; this further increases the difficulty of understanding the basis of the disease.

Enzyme replacement therapy (ERT) has been reported to reduce the accumulation of substrates in several tissues and thereby improve the clinical outcomes of patients with Fabry disease [7], [8], [9], [10]. Two different recombinant human GLAs (rhGLAs) are currently available for ERT. However, ERT often leads to the development of circulating immunoglobulin G (IgG) against rhGLA among male patients with Fabry disease, who completely lack the GLA protein, and these antibodies may cause allergic reactions and/or reduce the efficacy of ERT. In contrast, anti-GLA IgG only rarely develops in female patients with Fabry disease, in whom GLA is expressed in many cells [11], [12], [13], [14]. These results suggest that the circulating GLA protein is important for the production of anti-GLA antibodies. These results suggest that the endogenous GLA protein is important for prevention of anti-GLA antibody production due to recurrent rhGLA administration.

Considering these results, the measurement of the GLA protein in the blood is very important for understanding the pathogenetic basis of Fabry disease and issues of antibody production. However, few reports have been published on the measurement of the GLA protein in clinical samples, and all the reported methods involve enzyme-linked immunosorbent assay (ELISA), which is not highly sensitive [15], [16].

In this study, we developed a novel application of the highly sensitive immuno-polymerase chain reaction (PCR) assay method (designated as MUSTag for Multiple Simultaneous Tag) for measuring GLA protein levels in clinical samples and determined the GLA concentrations in serum and plasma from male patients with classic Fabry disease, female patients with Fabry disease, male patients harboring p.E66Q, and control subjects.

## Materials and Methods

This study involving human samples was approved by the Ethics Committee of Tokyo Metropolitan Institute of Medical Science and Meiji Pharmaceutical University. All participants and/or their parents provide their written informed consent to participate in this study. All raw data used in this study were summarized in Table S1.

### Reagents

**Figure 1:**
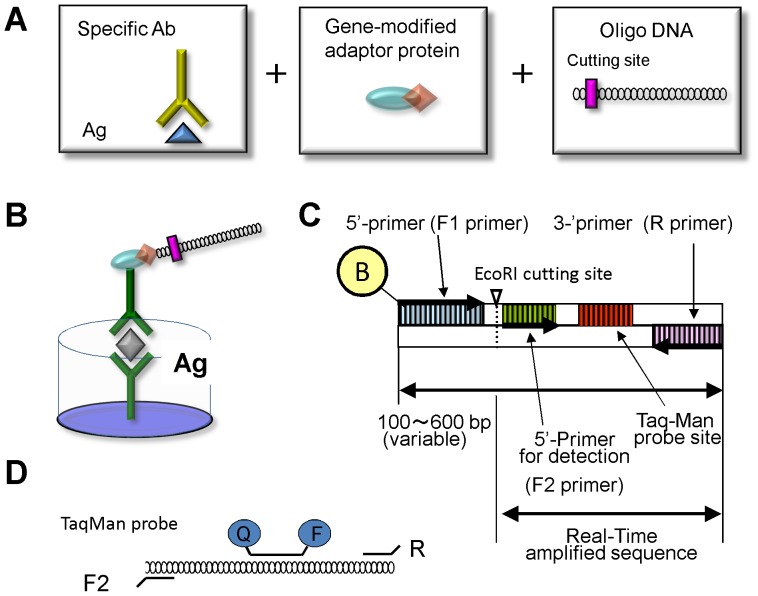
Structures of the immuno-polymerase chain reaction (PCR) Multiple Simultaneous Tag (MUSTag) components. **A.** The MUSTag assay consists of 3 parts: a capture antibody, a recombinant protein G-avidin fusion protein, and a synthesized biotin-conjugated oligonucleotide (A). **B.** The MUSTag assay is performed similar to the sandwich enzyme-linked immunosorbent assay (ELISA) but by using a biotin-labeled detection antibody instead of an enzyme-labeled antibody. **C.** MUSTag oligo-DNAs were designed to incorporate 5 defined sequence regions: the 5′ primer for template amplification with biotin labels on sequence (F1 primer) and the complement of the 3′ primer (R primer), the 5′ primer for qRT-PCR detection (F2 primer) with R primer, an *Eco*RI restriction site, and, for hybridization with fluorogenic TaqMan, a double-labeled hybridization probe that annealed between the F2 primer and the R primer. **D.** The complement sequences of the F2 and R primers and TaqMan Probe were used for qRT-PCR measurement of the original concentration of GLA.

The PCR reagents were obtained from Takara Bio, Inc. (Shiga, Japan). *Eco*RI was obtained from Nippon Gene Co., Ltd. (Tokyo, Japan). The F1, F2, and R primers (**Fig. 1**) and TaqMan-1were purchased from Operon Biotechnology, Inc. (Tokyo, Japan). Peroxidase-linked secondary antibodies were purchased from GE Healthcare (Buckinghamshire, UK). Sure Blue Reserves TMB microwell peroxidase substrate (1-Component) and TMB Stop Solution were purchased from Kirkegaard & Perry Laboratories, Inc. (Gaithersburg, ML). rhGLA (agalsidase beta) was purchased from Genzyme Japan (Tokyo, Japan).

### Patients and Samples

Serum and/or plasma samples for the determination of GLA protein level and activity were obtained from 10 male patients with classic Fabry disease (Male Fabry group, 9 and 10 serum and plasma samples, respectively, were collected), 10 female patients with Fabry disease (Female Fabry group, only plasma samples were collected), 12 male subjects harboring p.E66Q (E66Q group; c.196G>C nucleotide change, functional polymorphism), and 10 healthy controls (Control group; serum samples from 10 controls and plasma and leukocyte samples from 10 other 10 controls were collected). Their genotypes and levels of GLA activity in leukocytes are summarized in **Table 1**. This study was approved by the Ethics Committee of our institute.

**Table 1:** Genotype and α-galactosidase A (GLA) activity in leukocytes of patients with Fabry disease.

Group	Genotype	GLA activity
		(nmol/h·mg prot.)
Male Fabry 1*	p.G43V	<0.3
2	p.G195V	<0.3
3	p.R227X	<0.3
4	p.S345X	<0.3
5	p.W399X	<0.3
6	c.370del	<0.3
7	c.718-719del	<0.3
8	c.1277-1278del	<0.3
9	IVS5-1 g>c	<0.3
10	ND	<0.3
Female Fabry 11	p.G43V/WT	23
12	p.R112C/WT	2.5
13	p.G147E/WT	7.9
14	p.R227X/WT	24
15	p.W245X/WT	6.0
16	p.R301X/WT	21
17	p.Y356X/WT	0.6
18	c.215del/WT	37
19	c.409del/WT	14
20	c.1033-1034del/WT	5.2
E66Q	p.E66Q	23±9 [12] *
Control	WT	47±13 [10] *

ND: Not determined, WT: Wild-type.

* Mean ± SD [n].

### Design of the Immuno-PCR MUSTag Assay

The MUSTag assay method for measuring GLA protein levels in clinical samples was designed to combine the immune reaction between an antigen (GLA) and antibodies with PCR for signal enhancement (**Fig. 1**). In this assay system, the antigen reacts first with the capture antibody (provided by Keutzer J, Genzyme) and then with the detection antibody [17], which is linked to a 131-bp-long DNA oligonucleotide through a gene-modified adaptor protein, as described previously [18], [19], [20], [21]. After the immune reaction, the DNA oligonucleotide was removed from the detection antibody by restriction enzyme digestion, and the amount of the DNA was measured using quantitative real-time-PCR (qRT-PCR).

### Construction of Primers for PCR

Primers were designed according to an originally designated sequence (131-bp-long template) with the desired specificity and without homology to any mammalian genomes, a G+C content of <50%, defined duplex stability (T_m_, 55–60°C), and duplex internal stability for PCR primers, as described elsewhere [22]. The MUSTag assay involves DNA oligonucleotides designed to incorporate 5 defined sequence regions: a biotin-conjugated 5′ primer (F1 primer) for the labeling of the detection antibody; the complement of the 3′ primer (R primer) for amplification of the original template with F1 primer; the 5′ primer for qRT-PCR (F2 primer); an *Eco*RI restriction site; and for hybridization with fluorogenic TaqMan, a double-labelled hybridization probe that annealed between the F2 primer and the R primer (**Fig. 1**). Both the primers and a template sequence (131 bp) were designed to be free of duplex formation (dimers or hairpins). The sequences of the primers used were as follows:

F1∶5′-Biotin-CTTACTGGCTTATCGAAA-3′ and R: 5′-GGCAAGCCACGTTTGGTG-3′.

### MUSTag Plate Assay

Capture antibody (anti-GLA monoclonal antibody, 2 µg/mL, provided by KeutzerJ, Genzyme) in 50 mM sodium carbonate, pH 9.6, was added to each well of a 96-well Maxisorp microtiter plate (Nunc, Roskilde, Denmark) at 50 µL/well and incubated overnight (16 h) at 4°C for immobilization. Then, the antibody solutions were removed and the wells washed 3 times using the assay diluent/wash buffer (phosphate-buffered saline (PBS) with 0.05% Tween-20), with immediate aspiration of the buffer from the wells. The microtiter plate was inverted and slapped vigorously onto the absorbent material to remove the residual wash buffer. Non-adsorbed sites in the microtiter wells were blocked by incubation with 200 µL/well of PBS and 1% BSA for 1 h at room temperature. The blocking buffer was then removed and the wells washed 3 times. Next, 50-µL volumes of serial dilutions of each test antigen (serum, plasma, or standard rhGLA) were added to the capture antibody-coated wells of the microtiter plate. Negative control wells were filled with 50 µL of blocking buffer. The microtiter plate was incubated at room temperature for 1 h; the antigen solutions were removed; and the wells were washed 3 times, as before. The appropriately diluted MUSTag detection antibody (anti-GLA rabbit polyclonal antibodies) [17] conjugate was added to the test wells at 30 µL/well, and the microtiter plate was incubated at room temperature for 1 h. Then, the conjugate solutions were removed and the wells were washed 3 times. The bound DNA was released by adding 30 µL of *Eco*RI solution (10 UT/mL) to each well and incubated for 15 min at room temperature. The resulting supernatants were collected into 0.2-mL tubes. These supernatants contained the DNA oligonucleotide that had been used to label the MUSTag detection antibody.

The released DNA was measured by means of qRT-PCR using the MX-3005p quantitative real-time PCR (qRT-PCR) system (Stratagene, La Jolla, CA) to amplify and detect the oligonucleotides that were used to label the MUSTag detection antibody. The amplification reaction consisted of 10 µL of 2× FastStart Universal Probe Master with ROX reference dye (Roche Diagnostics, Mannheim, Germany), TaqMan Probe (Invitrogen, Carlsbad, CA) at a final concentration of 100 nM, F2 and R primers at a final concentration of 360 nM, and up to 17 µL of nuclease-free PCR-grade H_2_O in a final volume of 20 µL in 0.2-mL PCR tubes with dome-top lids. To each of these reactions was added 3 µL of the released DNA oligonucleotide in *Eco*RI solution. The thermal cycler was heated to 95°C for 10 min (initial activation step) for a hot start, and amplification was performed for 40 cycles using the following thermal cycling conditions: 95°C for 15 s for denaturation and 60°C for 60 s for annealing and extension.

Standard curves were calculated from the results of measuring rhGLA diluted to 10,000, 2,000, 400, 80, 16, 3.2, and 0.64 pg/mL in serum or plasma.

### MUSTag Beads Assay

For the MUSTag beads assay, the monoclonal anti-GLA capture antibody was conjugated to Tosyl-activated Dynabeadss M-280 (Invitrogen Dynal AS, Oslo, Norway) according to the manufacturer’s protocol. The MUSTag beads assay mixture, which included the anti-GLA capture antibody-conjugated magnetic beadss and the oligo-DNA-labeled anti-GLA detection antibody in an assay buffer containing 0.05% Tween-20, 0.45 M NaCl, 50 mM sodium phosphate (pH7.4), and 10% goat serum, was prepared and mixed with appropriately diluted samples (serum, plasma, or standard GLA) in a 96-well plate for 2 h at room temperature with shaking. The beads were captured for 3 min with a 96-well Magnetic-Ring Stand (Applied Biosystems, Foster City, CA) and washed 4 times with 200 µL/well of wash buffer (0.05% Tween-20, 0.5 M NaCl, and 20 mM Tris-HCl pH7.4). Then, the removal, amplification, and quantification of the DNA oligonucleotide were performed in the same manner as in the MUSTag plate assay.

For neglecting the background cross reaction of anti-GLA antibodies, we used anti-IL-6 antibody as a capture antibody in MUSTag plate assay, and measured the GLA concentrations in 10% serum from 10 healthy controls. The GLA concentration could be measured in the range from 341 pg/ml to 1,135 pg/ml without non-specific reactions (**Fig. 1**).

### Calculation of GLA Concentration

After the amounts of DNA oligonucleotide were measured by qRT-PCR, the Ct values for the DNA were calculated using MXPro QPCR Software version 3.20 (Agilent Technologies, Santa Clara, CA) according to the manufacturer’s protocol. The original concentration of GLA was calculated using a 4-parameter logistic nonlinear regression model in GraphPad Prism version 5.02 (Graph-Pad Software, Inc., San Diego, CA) as follows.

Equation for 4-parameter logistic nonlinear regression model.
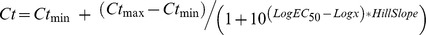



*Ct*_max_, Maximum asymptotic Ct value; *Ct*_min_, Minimum asymptotic Ct value; *EC*_50_, Inflection point value; X, the independent Ct value; and *Hill Slope*, the steepness of the curve. Coefficient of determination was obtained using simple regression analysis (Excel software).

### ELISA

ELISA-based measurement of the GLA protein concentrations in clinical samples was performed according to the standard method [15], [16]. The antibodies and standard used were the same as for the MUSTag plate and beads assay methods except that the detection was performed by horseradish peroxidase (HRP) activity using HRP-linked donkey anti-rabbit IgG. The immune reaction was analyzed using the microplate reader Benchmark (BIO-RAD) by adding peroxidase substrate (0.04% o-phenylenediamine, 0.6% H_2_O_2_, and 0.15 M citric acid buffer, pH 5.0) to each well.

### GLA Activity Assay

GLA activity levels in serum and plasma were determined fluorometrically using 4-methylumbelliferyl-α-D-galactopyranoside as the substrate and *N*-acetyl-d-galactosamine as an inhibitor of *N*-acetyl-α-d-galactosaminidase. The fluorescence was measured using a Wallac 1420 ARVO MX multilabel counter (Perkin Elmer, Waltham, MA) with excitation and emission wavelengths of 355 nm and 460 nm, respectively, as described previously [23].

### Statistical Analyses

The results are expressed as the mean and standard deviation (SD) or as individual data and subjected to 2-sided statistical analyses with EZR, which is a free software provided from Saitama Medical Center, Jichi Medical University, Japan [24]. The overall difference among the groups was assessed using the Kruskal Wallis test. If the Kruskal Wallis test indicated a significant difference, the p values of the differences among the groups were calculated using the Steel Dwass test. Asterisks were used to indicate p values of <0.05 (*), <0.01 (**), and <0.001 (***).

## Results

### Comparison of the Measurement of GLA Protein Levels Using ELISA and the MUSTag Plate and Beads Assays

**Figure 2:**
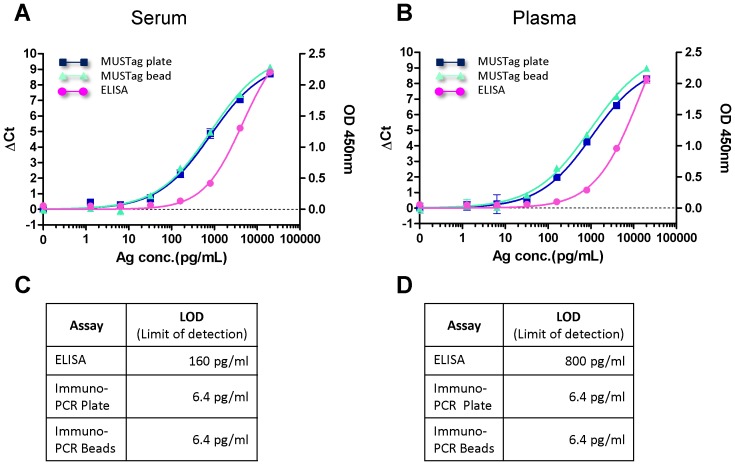
Comparison of sensitivities of MUSTag plate, beads assays, and ELISA for measuring α-galactosidase A (GLA). Standard curve of the GLA protein concentration in serum (**A**) and plasma (**B**). In each figure, closed boxes (▪), closed triangles (▴), and closed circles (•) indicate the results of the MUSTag plate assay, the MUSTag beads assay, and ELISA, respectively. **C.** Sensitivities of ELISA and the MUSTag plate and beads assays for measurement of the level of GLA protein in serum (**C**) and plasma (**D**).

We first compared the measurement of GLA protein levels using conventional ELISA and the MUSTag plate and beads assays. The samples consisted of rhGLA serially diluted into GLA-negative serum and plasma samples collected from Fabry patients, and the assays were optimized to yield a linear response over the biological range (**Fig. 2A and B**). We found that the maximal sensitivity (limit of detection, LOD) for GLA in serum to be 160 pg/mL for ELISA and 6.4 pg/mL for both MUSTag assays (plate and beads) (**Fig. 2C**). The LODs for GLA in plasma were 800 pg/mL for ELISA and 6.4 pg/mL for both MUSTag assays (plate and beads) (**Fig. 2D**). The MUSTag plate and beads assays were more sensitive than ELISA for the measurement of the GLA protein level.

### MUSTag Plate Assay for Measuring GLA Protein in Serum and Plasma

**Figure 3:**
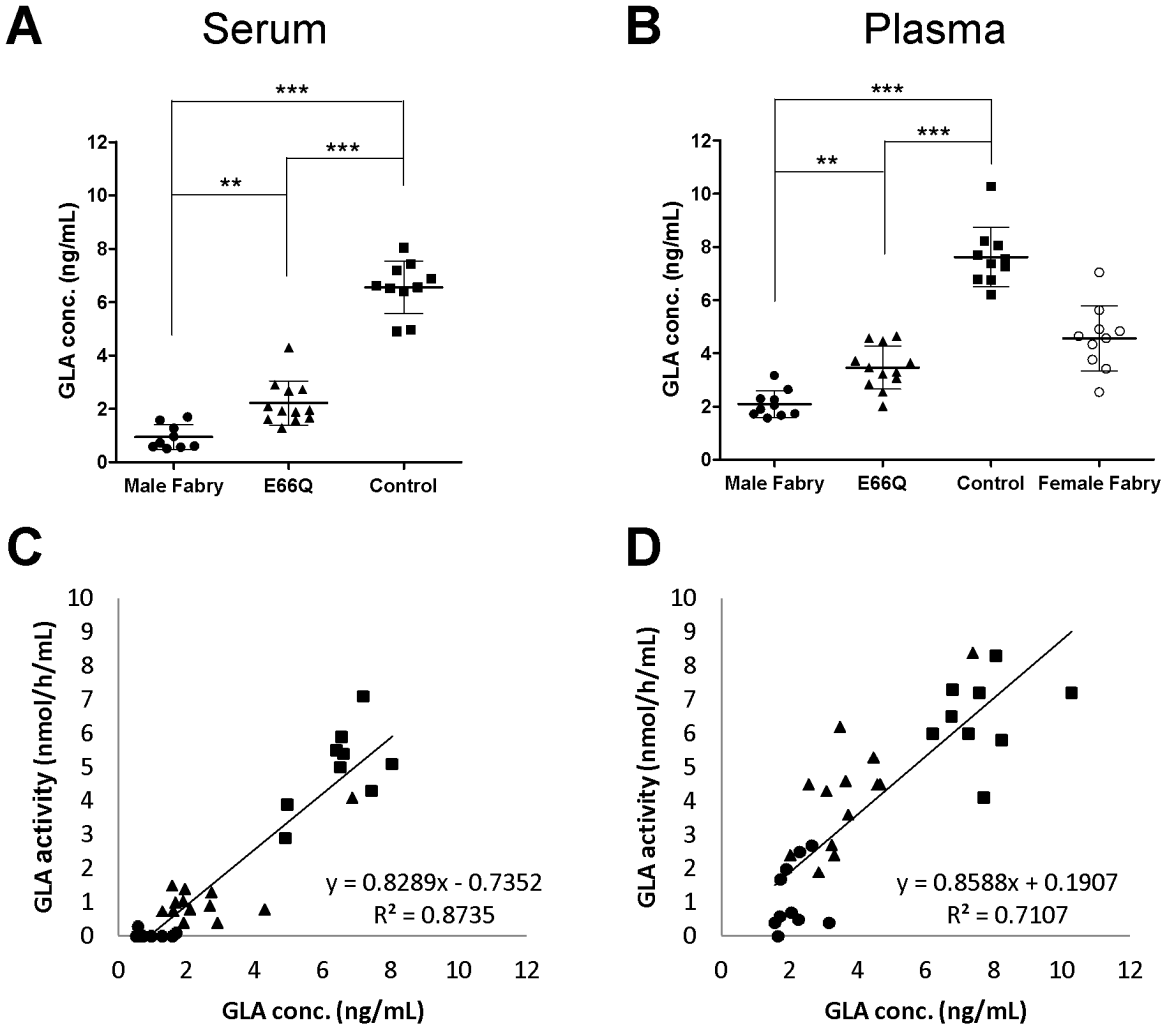
Measurement of the GLA protein level in serum/plasma using the MUSTag plate assay. **A.** The GLA concentrations in the serum samples collected from male patients with classic Fabry disease (Male Fabry), male subjects with p.E66Q (E66Q), and control subjects (Control). **B.** The GLA concentrations in the plasma samples collected from male patients with classic Fabry disease (Male Fabry), female patients with Fabry disease (Female Fabry), male subjects with p.E66Q (E66Q), and control subjects (Control). The statistically significant differences are indicated by asterisks: *p<0.05; **p<0.01; ***p<0.001. All experimental data are shown as the mean (bold horizontal bars) ± S.D. (error bars). **C.** The relationship between the enzymatic activity and protein level of GLA in serum. **D.** The relationship between the enzymatic activity and protein level of GLA in plasma. In each figure, closed circles (•), open circles (○), closed triangles (▴), and closed boxes (▪) show the data for samples from the Male Fabry, Female Fabry, E66Q, and Control groups, respectively.

To validate the MUSTag assay using clinical samples, the levels of GLA protein in serum and plasma from males with classic Fabry disease (Male Fabry), females with Fabry disease (Female Fabry), male subjects with p.E66Q (E66Q), and control subjects (Control) were measured using the MUSTag plate assay. The mean serum GLA protein levels were 0.95±0.46 ng/mL (n = 9), 2.22±0.83 ng/mL (n = 12), and 6.55±0.99 ng/mL (n = 10) in the Male Fabry, E66Q, and Control groups, respectively. The values of the Male Fabry, E66Q, and Control groups differed significantly from each other, as shown in **Fig. 3A** (Male Fabry vs. Control, p<0.001; E66Q vs. Control, p<0.001; Male Fabry vs. E66Q, p<0.01). The mean plasma GLA protein levels of the Male Fabry, Female Fabry, E66Q, and Control groups were 2.09±0.50 ng/mL (n = 10), 4.56±1.23 ng/mL (n = 10), 3.46±0.81 ng/mL (n = 12), and 7.62±1.12 ng/mL (n = 10), respectively. The values of the Male Fabry, E66Q, and Control groups differed significantly from each other, as shown in **Fig. 3B** (Male Fabry vs. Control, p<0.001; E66Q vs. Control, p<0.001; Male Fabry vs. E66Q, p<0.01). The Fabry Female group showed heterogeneity in the values.

**Figures 3C and 3D** show the relationships between the GLA protein level and the enzymatic activity in serum and plasma. The enzymatic activity correlated with the protein level in both serum (R^2^ = 0.8735) and plasma (R^2^ = 0.7107).

### MUSTag Beads Assay for Measuring the Levels of GLA Protein in Serum and Plasma

**Figure 4:**
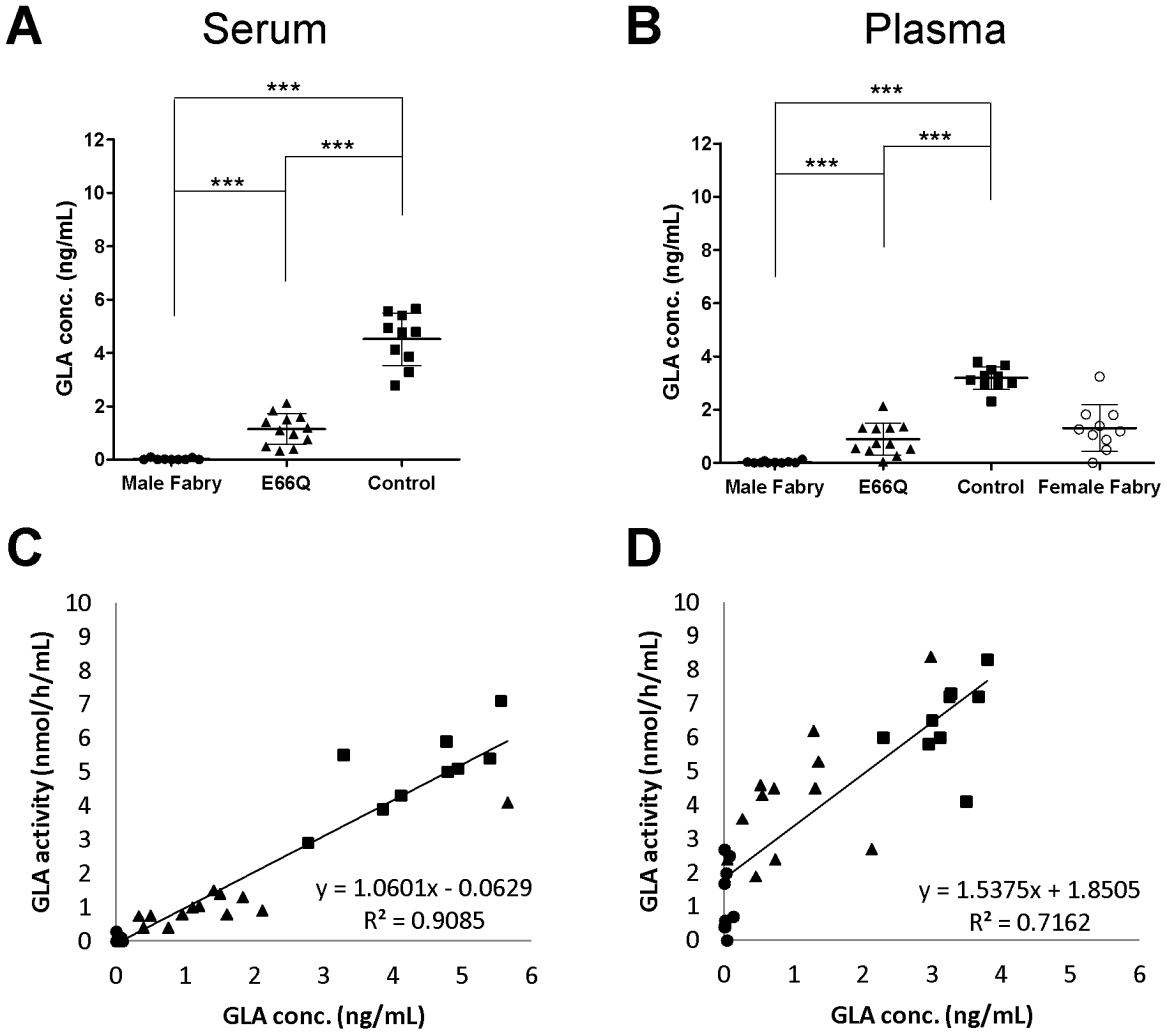
Measurement of the GLA protein level in serum/plasma using the MUSTag beads assay. **A.** The GLA concentrations in the serum samples from male patients with classic Fabry disease (Male Fabry), male subjects with p.E66Q (E66Q), and control subjects (Control). **B.** The GLA concentrations in the plasma samples collected from male patients with classic Fabry disease (Male Fabry), female patients with Fabry disease (Female Fabry), male subjects with p.E66Q (E66Q), and controls (Control). Statistically significant differences are marked with asterisks: *p<0.05; **p<0.01; ***p<0.001. All experimental data are shown as the mean (bold horizontal bars) ± S.D. (Error bars). **C.** The relationship between the enzymatic activity and protein level of GLA in the serum. **D.** The relationship between the enzymatic activity and protein level of GLA in the plasma. In each figure, closed circles (•), open circles (○), closed triangles (▴), and closed boxes (▪) show the data for samples from the Male Fabry, Female Fabry, E66Q, and Control groups, respectively.

We next measured the GLA protein levels in serum and plasma from the 4 groups using the MUSTag beads assay. The mean serum GLA protein levels of the Male Fabry, E66Q, and Control groups were 0.02±0.04 ng/mL (n = 9), 1.14±0.58 ng/mL (n = 12), and 4.51±0.98 ng/mL (n = 10), respectively. The mean plasma GLA levels of the Male Fabry, Female Fabry, E66Q, and Control groups were 0.03±0.04 ng/mL (n = 10), 1.31±0.88 ng/mL (n = 10), 0.89±0.59 ng/mL (n = 12), and 3.19±0.43 ng/mL (n = 10), respectively. The values of the Male Fabry, E66Q, and Control groups differed significantly from each other, as shown in **Fig. 4A and 4B** (serum, p<0.001; plasma, p<0.001). The Female Fabry group showed heterogeneity in the values. The results resembled those of the MUSTag plate assay, except that the measured values of all 4 groups were lower when the MUSTag beads assay was used, with the GLA protein level in the Male Fabry group typically being close to the LOD. The enzymatic activity levels in the serum and plasma correlated with the GLA protein levels (serum, R^2^ = 0.9085; plasma, R^2^ = 0.7162), as shown in **Fig. 4C and 4D**.

### ELISA for Measuring the Levels of GLA Protein in Serum and Plasma

**Figure 5:**
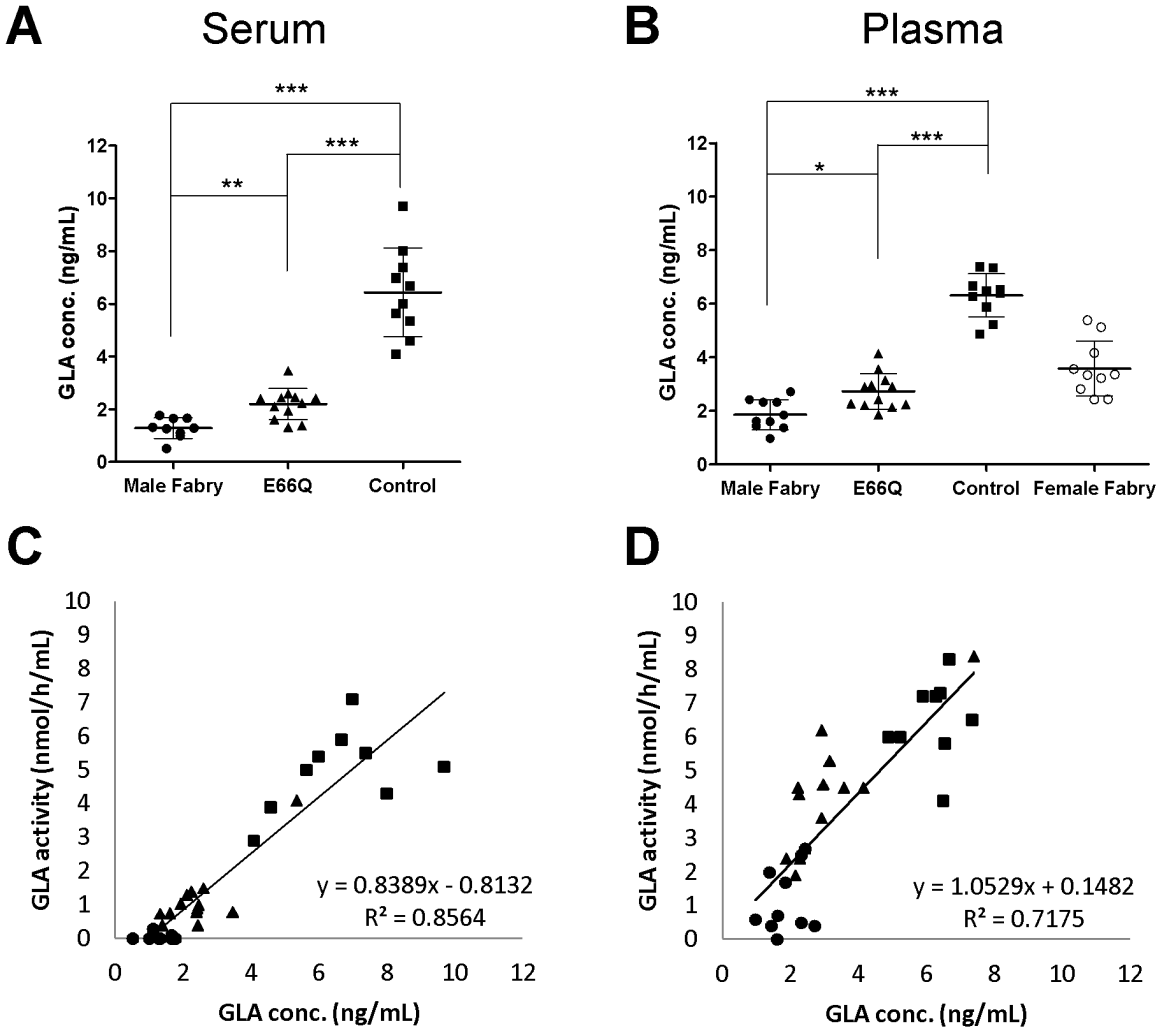
Measurement of the GLA protein level in serum/plasma using ELISA. **A.** The GLA concentrations in the serum samples collected from male patients with classic Fabry disease (Male Fabry), male subjects with p.E66Q (E66Q), and control subjects (Control). **B.** The GLA concentrations in the plasma samples collected from male patients with classic Fabry disease (Male Fabry), female patients with Fabry disease (Female Fabry), male subjects with p.E66Q (E66Q), and control subjects (Control). Statistically significant differences are marked with asterisks: *p<0.05; **p<0.01; ***p<0.001. All experimental data are shown as the mean (bold horizontal bars) ± S.D. (Error bars). **C.** The relationship between the enzymatic activity and protein level of GLA in the serum. **D.** The relationship between enzymatic activity and the protein level of GLA in the plasma. In each figure, closed circles (•), open circles (○), closed triangles (▴), and closed boxes (▪) show the data for samples from the Male Fabry, Female Fabry, E66Q, and Control groups, respectively.

Finally, we used ELISA to measure the GLA protein levels in serum and plasma samples collected from the 4 groups in order to compare the results of the MUSTag plate and beads assays with those of the conventional assay. The mean serum GLA protein levels of the Male Fabry, E66Q, and Control groups were 1.29±0.39 ng/mL (n = 9), 2.20±0.59 ng/mL (n = 12), and 6.44±1.67 ng/mL (n = 10), respectively. The values of the Male Fabry, E66Q, and Control groups differed significantly from each other, as shown in **Fig. 5A** (Male Fabry vs. Control, p<0.001; E66Q vs. Control, p<0.001; Male Fabry vs. E66Q, p<0.01). The mean plasma GLA protein levels of the Male Fabry, Female Fabry, E66Q, and Control groups were 1.86±0.56 ng/mL (n = 10), 3.58±1.03 ng/mL (n = 10), 2.73±0.66 ng/mL (n = 12), and 6.31±0.81 ng/mL (n = 10), respectively. The values of the Male Fabry, E66Q, and Control groups differed significantly from each other, as shown in **Fig. 5B** (Male Fabry vs. Control, p<0.001; E66Q vs. Control, p<0.001; Male Fabry vs. E66Q, p<0.05). The Female Fabry group showed heterogeneity in the values. These results are almost identical to those from the MUSTag plate assay. The enzymatic activity levels essentially correlated with the respective GLA protein levels in both serum (R^2^ = 0.8564) and plasma (R^2^ = 0.7175), as shown in **Fig. 5C and 5D**.

## Discussion

Antibody-based detection methods, such as ELISA and western blotting, are valuable tools for measuring the concentrations of target proteins, with high specificity and cost-efficiency. However, it is difficult to detect biological markers at concentrations less than 1–10 pg/mL, which is less than the LOD for ELISA. To improve upon the sensitivity of traditional antibody-based methods for protein detection, a modified immuno-PCR method, MUSTag, was developed and has been continually refined [18], [19], [20], [21], [22], [23], [25], [26], [27], [28]. The MUSTag method can be used to detect multiple protein targets simultaneously [29], [30] by labeling each antibody with a specific 5′-biotinylated DNA oligonucleotide by indirect linkage to a recombinant protein G/avidin fusion protein and detecting the DNA oligonucleotides from each antibody separately using qRT-PCR with the TaqMan® probe.

In this study, we modified the immuno-PCR method and developed novel MUSTag plate and beads assays for measuring the levels of GLA protein in serum and plasma. First, we compared the sensitivities of these methods with that of ELISA, by using rhGLA as a standard and found that the MUSTag assays could detect as little as 6.4 pg/mL of GLA, making them apparently more sensitive than ELISA. Then, we measured the GLA protein levels in serum and plasma from male patients with classic Fabry disease, female patients with Fabry disease, male subjects with p.E66Q, and control subjects. p.E66Q is a functional polymorphism that has been identified with unexpectedly high frequency in Japanese and Korean populations during screening for Fabry disease by using dry blood spots and serum/plasma samples with long incubation times [4], [6]. Biochemical studies revealed that p.E66Q destabilizes the GLA protein, and subjects harboring the E66Q amino acid substitution exhibited relatively high residual enzyme activity in leukocytes when the incubation time was kept short. None of the tissue samples obtained from subjects with p. E66Q exhibited either accumulation of Gb3 or any pathological changes. In the present study, the MUSTag plate and beads assay as well as ELISA could differentiate between the Fabry Male, E66Q, and Control groups. As expected, female patients with Fabry disease exhibited heterogeneous GLA protein levels in serum and plasma irrespective of the method used, which is consistent with the X-chromosomal inheritance of the disease. The GLA protein levels were lower when measured using the MUSTag beads assay than when using the MUSTag plate assay or ELISA, and the GLA protein levels of the Fabry Male group were close to the LOD. Since the Male Fabry group examined herein includes cases with deletions (c.718–719del, c.370del, and c.1277–1287), nonsense mutations (p.R227X, S345X, and p.W399X), a splicing mutation (IVS5-1 g>c), and a missense mutation (p.G43V) leading to a large structural change in the core of the molecule, it is thought that the blood GLA protein levels from these subjects are deficient or very low. Therefore, the values obtained using the MUSTag beads assay should reflect the correct GLA concentrations in blood with minimal or no background noise, and this assay method is expected to be particularly useful for identifying quantitative defects in the GLA protein in Fabry disease.

ERT by exogenous administration of lysosomal enzymes is currently successfully employed for the treatment of lysosomal storage diseases, including Fabry disease. However, recurrent administration of the recombinant enzymes to patients who do not produce any endogenous enzymatic proteins may lead to the release of antibodies against the enzymes [11], [12], [13], [14]. Anti-GLA antibodies have been reported to cause allergic reactions and reduction of the efficacy of ERT in patients with Fabry disease [31], [32], [33], [34]. The antibody production frequently occurs in male patients with Fabry disease but not in female patients with Fabry disease. This suggests that the antibody production in male patients with Fabry disease may be associated with the deficiency of GLA protein. To verify the hypothesis, it is important to examine the blood GLA protein level prior to initiating ERT and monitor the antibody production after ERT. The MUSTag beads assay could be available for this purpose.

In conclusion, we developed a novel application of the immuno-PCR assay method, MUSTag, for measuring the GLA protein levels in serum and plasma. This method is expected to be useful as a sensitive method for the determination of GLA level in blood and elucidation of the pathogenetic basis of Fabry disease.

## Supporting Information

Figure S1 **For evaluation of nonspecific reaction of MUSTag beads method, we used an antibody against IL-6 (MUSTag GLA/IL-6) as a capture antibody instead of the antibody against a-galactosidase (MUSTag GLA/GLA). A:** Measurement of GLA protein levels with MUSTag GLA/GLA and MUSTag GLA/IL-6 in each 10% serum of health control (n = 10). Average concentration (pg/ml), Standard deviation, and % coefficient of variation are shown as AVE. Conc., SD, and %CV, respectively. **B:** The results were shown in the graph. We can neglect the background values in the assay with MUSTag GLA/IL-6.
(TIF)

Table S1 **All raw data of measurement of GLA protein and the activity in this manuscript.**
(XLSX)
