# Supplementary figures and images for: Development of a Highly Sensitive Immuno-PCR Assay for the Measurement of α-Galactosidase A Protein Levels in Serum and Plasma
Source: PLoS One. 2013 Nov 13;8(11):e78588. doi: 10.1371/journal.pone.0078588 (PMC3827252; doi:10.1371/journal.pone.0078588)

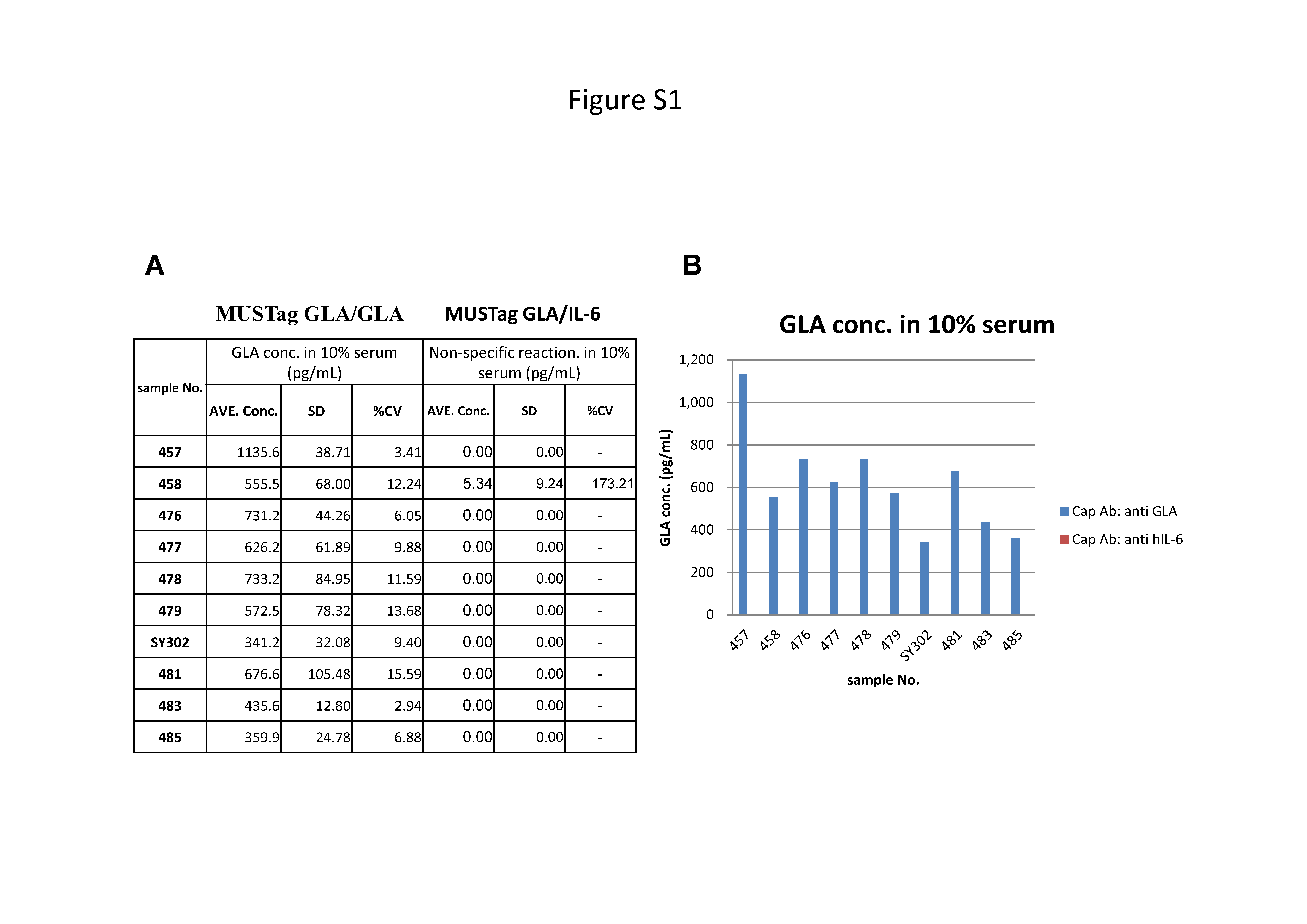

Supplement: Figure S1 — For evaluation of nonspecific reaction of MUSTag beads method, we used an antibody against IL-6 (MUSTag GLA/IL-6) as a capture antibody instead of the antibody against a-galactosidase (MUSTag GLA/GLA). A: Measurement of GLA protein levels with MUSTag GLA/GLA and MUSTag GLA/IL-6 in each 10% serum of health control (n = 10). Average concentration (pg/ml), Standard deviation, and % coefficient of variation are shown as AVE. Conc., SD, and %CV, respectively. B: The results were shown in the graph. We can neglect the background values in the assay with MUSTag GLA/IL-6. (TIF) [file pone.0078588.s001.tif]
